# Making Sense of Bullying: Brooding and subsequent sleep problems in seemingly safe work environments

**DOI:** 10.1186/s40359-026-04606-2

**Published:** 2026-04-28

**Authors:** Michael Rosander, Morten Birkeland Nielsen

**Affiliations:** 1 Department of Behavioural Sciences and Learning, Linköping University, Campus Valla, Linköping, 581 83 Sweden; 2 National Institute of Occupational Health, Oslo, Norway; 3 Department of Psychosocial Science, University of Bergen, Bergen, Norway

**Keywords:** Workplace harassment, Insomnia symptoms, Rumination, Sense-making, Hostile work climate

## Abstract

**Background:**

Work and sleep mutually influence one another: adequate sleep supports cognitive functioning and social regulation at work, whereas safe and respectful working conditions facilitate recovery and sleep. Although workplace bullying has been proposed as an important work-related determinant of sleep problems, the empirical base has been dominated by cross-sectional research, with comparatively few longitudinal studies. Furthermore, the mechanisms underlying this association, as well as the potential reverse association, remain poorly understood.

**Methods:**

Using a large longitudinal national probability sample of the Swedish workforce (*N* = 2,024), the present study examined how exposure to bullying is associated with sleep problems, and whether sleep problems, in turn, increase vulnerability to subsequent bullying, in both directions through cognitive and behavioural mechanisms, and how these processes depend on the work environment.

**Results:**

Exposure to bullying was indirectly related to subsequent sleep problems via increased brooding, with this pathway most pronounced in seemingly safe work environments. In the reversed direction, sleep problems were indirectly related to later exposure to bullying via brooding and conflict involvement; the brooding pathway was evident only in more hostile work environments, whereas the conflict involvement pathway operated across levels of hostile work climate.

**Conclusions:**

Taken together, the findings indicate that the relationship between bullying and sleep is neither simple nor uniformly reciprocal. Instead, different work environments intensify risk in different directions, highlighting the importance of cognitive sense-making processes and organizational context in understanding when and how bullying and sleep problems become intertwined.

**Supplementary Information:**

The online version contains supplementary material available at 10.1186/s40359-026-04606-2.

## Introduction

Work and sleep are closely interwoven. Sleep is essential for cognitive functioning, emotional stability, and the ability to meet daily demands at work, whereas poor sleep may impair the cognitive and social capacities needed to perform work tasks optimally and to navigate interpersonal interactions. At the same time, conditions at work may undermine the possibility of obtaining sufficient, high-quality sleep, as many employees continue to think about work long after leaving it. Consequently, work and sleep mutually influence one another, with good sleep being a requirement for functioning at work, whereas safe and respectful working conditions are a prerequisite for good sleep. Supporting this argument, work-related stress has frequently been identified as a central correlate of sleep problems [1].

As for specific working conditions that influence sleep, emerging evidence suggests that workplace bullying—that is, systematic and repeated exposure to negative behaviours at work from other employees, where the ability to stop the treatment becomes increasingly difficult the longer it persists [2, 3]—is likely to be an important determinant of sleep problems [4], with some studies also pointing to a potential reverse effect of sleep on the risk of bullying [5]. However, the mechanisms linking exposure to bullying and sleep remain poorly understood [4]. As individuals are motivated to make sense of their experiences, stressful work events—particularly negative social interactions—are often cognitively processed after work, involving sustained sense-making efforts to understand what has occurred [6]. Accordingly, sense-making processes that involve brooding over negative events represent one plausible mechanism through which workplace bullying may affect sleep.

Bullying behaviours are often indirect and ambiguous in nature and may therefore be difficult for those exposed to interpret [7]. Because sense-making involves attempts to organize discrepant or unexpected events and place them within a meaningful framework that allows individuals to understand what is happening and anticipate what might occur next [8], exposure to bullying behaviours is likely to trigger sustained efforts to make sense of the experience. In the context of workplace bullying, this may involve attempts both to understand why one has been singled out and to identify possibilities for escaping or counteracting the behaviour, with the latter becoming increasingly limited as the negative treatment persists and power imbalances grow [9, 10]. Brooding may be viewed as a sense-making process that does not arrive at a plausible solution but instead keeps delving into perceived possibilities and impossibilities—a form of sense-making that, in a sense, makes very little sense. Or, as Weick [8] noted, “accuracy is nice, but not necessary” [8]. Brooding has been shown to be strongly associated with impaired recovery and sleep problems [11, 12].

Specifically, the aims of the present study are to examine how exposure to workplace bullying affects brooding and, in turn, sleep problems, and whether these processes depend on the broader work environment. The study also investigates a potential reverse effect, whereby sleep problems may increase the risk of becoming bullied through two proposed mechanisms, as well as the role of the work environment in shaping these associations.

### The association between bullying and sleep

Although workplace bullying is often simplified into dichotomous categories such as victims/targets and non-victims/non-targets, it is intended to capture a complex and gradually escalating process rather than an either–or phenomenon [13, 14]. Bullying is a cyclical and cumulative process involving the gradual build-up of negative behaviours that lead to a power imbalance between target and perpetrator in work environments where such behaviour is normalized or goes unchallenged [3]. While bullying behaviours may range from subtle and indirect to overt aggression, the early stages are often characterised by isolated and ambiguous incidents—such as social exclusion or the spreading of rumours—which can make it difficult for the target to understand what is happening. As the negative treatment accumulates, it may eventually become clearer that one is being exposed to bullying, but prior to this point the experience may be perceived as highly unusual and difficult to make sense of.

Previous studies have shown an association between exposure to bullying and sleep problems, and a systematic review and meta-analysis reported that targets of bullying had a more than twofold risk of sleep problems [4]. However, the same review identified substantial methodological limitations and knowledge gaps in the existing literature. Most studies relied on cross-sectional designs, precluding conclusions about temporal ordering and the direction of the associations. Among the few prospective studies available, all but one used single-item measures of bullying—an approach that has been discouraged because it provides no information about the nature of the exposure, including distinctions between subtle and overt behaviours [15]. Moreover, most prospective studies employed long time frames (24–84 months), which may be ill-suited for capturing sleep problems as a proximal outcome of bullying exposure. In addition, only three studies examined a potential reverse effect of sleep problems on subsequent bullying.

Since the systematic review by Nielsen, Harris [4], a few additional studies on bullying and sleep have been conducted. Using diary designs with time frames ranging from a few days to several months, Rodriguez-Muñoz and colleagues [16–19] showed that sleep problems following exposure to bullying tend to develop gradually and accumulate over time, suggesting the need for longitudinal designs—though not necessarily with multi-year lags. In addition, Nielsen, Pallesen [20] reported prospective findings from a national probability sample with a six-month time lag. Using a behavioural experience method to assess bullying, they found that exposure to bullying was associated with subsequent changes in sleep problems.

Taken together, evidence for an association between bullying and sleep problems relies largely on cross-sectional data, and the prospective studies that do exist suffer from methodological limitations that restrict their interpretability. As a result, evidence regarding causal associations remains mixed and inconclusive, underscoring the need for further longitudinal research. In addition, existing studies have rarely examined the mechanisms linking bullying and sleep problems, or moderators that may clarify when and for whom these associations are most pronounced [4]. With the exception of a small number of longitudinal diary studies [16, 17, 19], research on mechanisms has primarily relied on cross-sectional designs (e.g., [12, 21, 22]), limiting conclusions about temporal ordering and underlying processes [23]. However, as cross-sectional research may provide useful theoretical starting points [24], a noteworthy finding across such studies is that rumination emerges as a key mediator.

### Brooding as a mediator

Theoretically, the association between exposure to bullying and sleep problems can be understood as an affective–cognitive process in which repeated negative interpersonal events elicit emotional reactions and sustained cognitive activation that extend beyond working hours and interfere with recovery. Such activation increases the likelihood of engaging in repetitive negative thinking, of which rumination represents one central indicator. Rumination refers to conscious, repetitive thoughts centred on a common instrumental theme that occur in the absence of immediate external demands [25] and is typically described as a negative process involving passive and repetitive thinking about adverse experiences [26]. It is commonly conceptualized as comprising two components: *reflective pondering*, which is more analytical and problem-oriented, and *brooding*, which is more affective and passive and involves dwelling on negative experiences [27, 28]. Brooding has also been described as affective rumination [29] or as a form of work-related perseverative cognition [30]. Exposure to bullying behaviours often involves helplessness, uncertainty, and a perceived lack of control [10, 31]—conditions that are particularly conducive to affective brooding. Accordingly, exposure to workplace bullying may trigger intense rumination and worry [17, 32], which in turn have been linked to reduced well-being, including anxiety, depression, fatigue, and PTSD [33]. In the present study, we focus on the affective component of rumination, brooding. For the sake of clarity, the term brooding is used throughout the remainder of the paper when referring to this form of repetitive negative thinking, except where it is necessary to refer specifically to rumination as the broader construct examined in previous research.

Affective events theory (AET; [34]) provides a useful framework for understanding why exposure to workplace bullying can become so psychologically taxing that it affects sleep. According to AET, employees’ emotional reactions arise from discrete work events appraised as positive or negative, and these affective reactions—rather than the events themselves—drive immediate behavioural tendencies and shape more enduring attitudes over time. Bullying behaviours can therefore be understood as recurrent negative events that repeatedly trigger adverse affective responses, which may become amplified as employees attempt to make sense of their experiences and spill over into cognitive processes such as brooding. AET further implies that these affective and cognitive responses extend beyond the workplace, interfering with recovery and sleep and thereby increasing vulnerability to further negative affective reactions. In this way, bullying may set in motion a cycle of emotional strain and impaired sense-making that becomes increasingly difficult to interrupt.

The cognitive activation theory of stress (CATS; [35]) further clarifies why repeated exposure to negative social events, such as bullying behaviours, can generate sustained strain. According to CATS, stress reactions are driven not only by the objective characteristics of a stressor but by how individuals appraise its controllability and predictability. When situations are perceived as manageable, physiological activation supports adaptive coping; however, when stressors are appraised as uncontrollable—as is typical in bullying situations—activation becomes prolonged and maladaptive. Over time, such sustained activation may foster helplessness, heighten negative affect, and impair cognitive functions essential for effective sense-making, including concentration, planning, and problem solving. In the context of workplace bullying, CATS thus highlights how repeated exposure to uncontrollable interpersonal stressors can maintain chronic activation that extends beyond working hours, undermining recovery and sleep and increasing vulnerability to further emotional and cognitive strain.

Taken together, AET and CATS provide complementary insights into why bullying may trigger brooding and, ultimately, sleep problems. AET highlights how repeated negative interpersonal events evoke adverse affective reactions that continue to occupy cognitive resources after work, thereby creating fertile ground for repetitive negative thinking. CATS, in turn, explains why these affective and cognitive processes are particularly likely to become prolonged in bullying situations, where stressors are recurrent and largely uncontrollable, leading to sustained activation that impairs recovery.

These perspectives are consistent with empirical studies showing that rumination-related processes mediate the association between exposure to bullying behaviours and impaired sleep. Prior research, although mainly based on cross-sectional designs and often examining constructs that only partially capture rumination, has demonstrated partial or full mediation through constructs such as negative work rumination [12], affective rumination [22], worrying [36], mental distress [21, 37], anxiety [16], and anger rumination [17, 19]. The time frame also appears to be important. Pereira, Meier [38] found no indirect effect of worrying on sleep over a two-week period; however, longitudinal diary studies suggest that rumination-related mechanisms may unfold gradually over time [17, 19].

Because much of the existing research relies on cross-sectional designs and single-item measures of bullying, conclusions about temporal ordering and underlying mechanisms remain limited. In addition, to fully understand the association between exposure to bullying and brooding, individual differences must be taken into account. Neuroticism has been associated with both exposure to bullying [39–41] and a greater tendency to engage in brooding [27]. At the same time, controlling for dispositional traits in stress–response models may raise concerns about over-controlling, as such traits may partly reflect the processes through which individuals respond to stressful experiences [42]. While baseline brooding captures prior levels of the focal cognitive response, neuroticism reflects broader dispositional negative emotionality that has been linked to both victimization experiences and brooding-related processes. Only one previous study has accounted for such dispositional influences: Rodriguez-Muñoz, Notelaers [36] controlled for negative affectivity in a cross-sectional study of bullying and sleep, with worrying as the mediator, showing significant associations with bullying, worry, and lack of sleep.

To address these issues, the present study includes neuroticism as a conservative control while also examining whether the results are robust to models excluding this trait. The study draws on a longitudinal national probability sample and employs a recommended behavioural measure of workplace bullying [15], allowing for a more rigorous test of whether brooding mediates the association between bullying and subsequent sleep problems beyond effects attributable to baseline levels of brooding and sleep problems. On this basis, we propose the following:

- Hypothesis 1: Exposure to bullying behaviours at baseline will be positively associated with an increase in sleep problems at follow-up, and this association will be mediated by an increase in brooding, controlling for baseline levels of neuroticism, brooding, and sleep problems.

### The role of the work environment

It is well established that the work environment influences the occurrence of bullying [43]. According to the work environment hypothesis, organizational deficiencies constitute primary drivers of bullying [44]. A related but less examined question is whether the work environment also shapes how individuals perceive and interpret bullying. We argue that it does.

Work environments characterized by distrust, tension, and conflict provide fertile ground for bullying [45]. Such hostile climates may generate frustration and interpersonal frictions that, if not properly managed, escalate into bullying [46] and risk becoming normalized in everyday interactions [10]. When negative treatment is widespread—even if only few employees are exposed to systematic bullying—the target may stand out less, both to others and to themselves, than in seemingly friendly workplaces with low levels of hostility. By contrast, exposure to bullying behaviours in a seemingly friendly environment is more likely to be perceived as unexpected and difficult to comprehend, particularly as bullying often begins with indirect and passive behaviours that may be challenging to recognize and make sense of [13].

From a trauma perspective, such experiences may threaten fundamental assumptions about the world as coherent, predictable, and meaningful, as well as about oneself as competent and valued [47]. This can increase cognitive load and intensify efforts to make sense of what appears to be a senseless situation, thereby amplifying brooding. From a CATS perspective [35], reactions to stressors depend on how individuals interpret their controllability and predictability; when negative treatment is perceived as inexplicable or uncontrollable, activation is more likely to become prolonged and difficult to regulate. Such sustained activation may hinder effective coping and increase the likelihood of persistent brooding, which in turn can interfere with recovery and sleep. Thus, we propose the following:

- Hypothesis 2: The indirect effect of exposure to bullying behaviours at baseline on the increase in sleep problems at follow-up via an increase in brooding will depend on the level of hostile work climate, such that the indirect effect will be weaker when hostile work climate is higher and stronger when it is lower, controlling for baseline neuroticism, brooding, and sleep problems.

### A reverse effect

So far, we have argued that exposure to bullying may lead to sleep problems, particularly in seemingly friendly work environments. An equally relevant question is whether sleep problems may, in turn, increase the risk of subsequent bullying. Only a few studies have examined this possibility, and their findings remain inconclusive [4]. Hansen, Hogh [5] reported an increased risk of later bullying among employees with difficulties waking up, whereas Johannessen and Sterud [48] and Vedaa, Krossbakken [49] found no such effects. Notably, although bullying may be a long-lasting phenomenon [41], the long time lags used in these studies—ranging from two to four years—may have reduced the likelihood of detecting meaningful change.

For sleep problems to predict subsequent exposure to bullying, their consequences must manifest in work-related behaviour and daily functioning. Impaired sleep is known to affect cognitive functioning and attentional control, including slower responses, reduced sensitivity to stimuli, and more frequent lapses in attention, which may translate into observable tiredness during the workday (cf. [50, 51]). Consistent with this, Rosander and Nielsen [52] showed that feeling tired at work is a risk factor for subsequent bullying.

Theoretically, the link between cognitive impairments resulting from poor sleep and later exposure to bullying can be understood from a social interactionist perspective [53]. Tiredness at work may make it difficult to meet expectations regarding productivity or social conduct, leading to behaviour that is perceived as deviating from workplace norms and as requiring correction. As argued by Tedeschi and Felson, such deviations may elicit negative reactions aimed at enforcing performance or social standards, which—if left unmanaged—may escalate into bullying. This process may manifest as increased involvement in conflicts, thereby providing one mechanism through which sleep problems increase the risk of subsequent bullying.

It is also possible that the cognitive consequences of poor sleep alter how employees perceive their work environment and the behaviours they encounter. According to the gloomy perception mechanism [54], impaired cognitive functioning and reduced emotional and attentional regulation may lead individuals to interpret ambiguous or mildly negative events as more severe than they actually are. This may manifest as increased brooding, thereby providing another mechanism through which sleep problems may elevate the risk of bullying—or at least the interpretation of workplace events as bullying behaviours. When tired, employees may become more sensitive to social cues and perceive everyday interpersonal frictions as signs of more serious mistreatment, even when the objective behaviour has not changed. Regardless of the underlying mechanism, the outcome is an increased likelihood of perceiving oneself as bullied, and it is this subjective perception that most strongly affects the target [10]. Thus, we propose the following:

- Hypothesis 3: Sleep problems at baseline will be positively associated with an increase in exposure to bullying behaviours at follow-up, and this association will be mediated by (a) an increase in conflict involvement, and (b) an increase in brooding, controlling for baseline levels of exposure to bullying behaviours, brooding, conflict involvement, and neuroticism.

The reverse effect may also depend on the work environment. In hostile work environments, the risk of exposure to bullying is generally higher [45], whereas more well-organized workplaces are typically better equipped to handle deviations from expected behaviour in constructive ways, thereby reducing the risk that such deviations escalate into bullying [55]. Against this background, we propose the following:

- Hypothesis 4: The indirect association between sleep problems at baseline and subsequent exposure to bullying behaviours will be moderated by the work environment, such that the indirect effects via (a) increased conflict involvement and (b) increased brooding will be stronger in work environments characterized by higher levels of hostile work climate, controlling for baseline levels of exposure to bullying behaviours, brooding, conflict involvement, and neuroticism.

## Methods

The study used a longitudinal probability sample drawn from the Swedish working population aged 18 years and older, restricted to employees in workplaces with at least ten staff members. Statistics Sweden, the national statistical agency, managed the sampling procedure and all participant contact. Baseline data were collected in autumn 2024 (*N* = 3,307), with follow-up data collected approximately seven months later in spring 2025. In total, 2,024 individuals participated at both waves. For each wave, participants received study information and consent materials via their digital mailbox or, when unavailable, by post. Following data collection, Statistics Sweden appended register information from the Swedish National Population Register (e.g., biological sex, age, and educational level), anonymised the dataset, and provided it to the research team. The study was approved by the Swedish Ethical Review Authority (Protocol No. 2023-06603-01).

### Participants

The sample comprised 58% women and 42% men, with a mean age of 49.44 years (SD = 10.50). On average, they had worked at their current workplace for 10.11 years (SD = 9.62), and most held a permanent position (96%). The majority were born in Sweden (89%). Regarding educational attainment, 16% had completed compulsory school, 33% upper secondary education, and 51% held a university degree.

### Measures

*Workplace bullying* was measured using the Swedish version of the Negative Acts Questionnaire–Revised (NAQ–R; [56, 57]), comprising 22 items describing negative and unwelcome behaviours at work. Participants reported how often they had experienced each behaviour during the past six months on a five-point frequency scale ranging from *never* to *daily*. Internal consistency was high at both measurement points (Cronbach’s α = 0.89 at T1 and T2).

*Brooding* was assessed with three items capturing the affective component of work-related rumination, consistent with Frone’s [26] conceptualisation of negative work rumination (e.g., “I often brood over things that have happened during the workday even when I’m at home”). Internal consistency was good (α = 0.87 at T1 and T2).

*Sleep problems* were measured as distress related to difficulties with falling asleep, maintaining sleep, and waking up too early [58]. Responses reflected the degree of distress associated with each difficulty (0 = *no distress* to 3 = *severe distress*). Internal consistency was acceptable (α = 0.76 at T1 and T2).

*Conflict* *involvement* was assessed with a single item asking: “How often have you experienced difficulties cooperating, tensions, or been in conflict with one or more people at your workplace during the past six months?”. Responses were given on a five-point scale ranging from *never* to *very often*.

*Hostile work climate* (HWC) was measured using a five-item scale capturing the full range from a safe and supportive work environment to a hostile one [45, 59]. Two items reflected a positive and safe climate (e.g., “At our workplace, the atmosphere is good”) and were reverse-coded, whereas three items captured the opposite pole of the construct (e.g., “My workplace is characterised by mistrust, conflicts, misunderstandings, and malice”). Internal consistency was high (α = 0.88 at T1 and T2).

Brooding, sleep problems, and HWC were measured using a seven-point Likert scale.

Sex and age were included as control variables because both have been linked to workplace bullying [60, 61] and to variation in brooding [62, 63]. Neuroticism was likewise included as a control variable, given its established associations with brooding [64] and bullying [41]. Neuroticism was assessed using four items from the neuroticism subscale of the Mini-IPIP [65], based on the Five-Factor Model of personality [66]. Responses were provided on a seven-point Likert scale, and internal consistency was acceptable (Cronbach’s α = 0.76 at T1).

### Data analysis

Data were analysed using structural equation modelling in Stata 19.5 with maximum likelihood estimation. Unstandardized coefficients (*b*) are reported throughout, and tables additionally report standardized coefficients (β) to facilitate interpretation of effect sizes. The analyses were estimated as path models within a structural equation modelling framework, allowing the longitudinal relations between variables, including mediation, moderation, and autoregressive paths, to be estimated simultaneously within a single model. To test Hypothesis 1, we estimated a longitudinal mediation model, and Hypothesis 2 was tested by extending this model to include HWC as a moderator. Hypotheses 3 and 4 were examined using a reversed moderated mediation model with two mediators and HWC as moderator of both indirect effects. The mediators and moderator were mean-centred. All models adjusted for baseline levels of the mediators and outcomes, as well as sex, age, and neuroticism. Because baseline levels of the mediators and outcomes were included in the models, the estimated effects reflect increases or decreases in these variables between T1 and T2. Structural models were estimated using listwise deletion. Missing data were minimal, resulting in the exclusion of between 5 and 12 cases depending on the model.

Prior to the analyses, the data were inspected for extreme outliers and potential violations of model assumptions. No indications of problematic multicollinearity or other violations affecting model estimation were observed. Because exposure to bullying behaviours is typically skewed, indirect and conditional indirect effects were estimated using bootstrapping with 5,000 resamples, which reduces reliance on distributional assumptions [67), and 95% percentile bootstrap confidence intervals (BootCI) are reported. The Johnson–Neyman technique was used to identify regions of significance. The index of moderated mediation was calculated to assess whether indirect effects depended on the moderator.

## Results

Table 1 presents means, standard deviations, and intercorrelations among the study variables. As expected, the central study variables were positively associated, and the constructs showed clear stability over time.

**Table 1:** Descriptive statistics of the study variables

Variables	Mean	SD	1.	2.	3.	4.	5.	6.	7.	8.	9.	10.	11.	12.
1. Sex	0.57	0.49	–											
2. Age	49.44	10.50	.01^ns^	–										
3. Neuro (T1)	3.22	1.09	0.18	− 0.21	–									
4. NAQ (T1)	1.22	0.30	0.10	− 0.08	0.30	–								
5. NAQ (T2)	1.21	0.30	0.09	− 0.10	0.27	0.68	–							
6. HWC (T1)	2.42	1.14	0.10	− .02^ns^	0.33	0.61	0.49	–						
7. HWC (T2)	2.44	1.15	0.12	− 0.05*	0.32	0.51	0.61	0.71	–					
8. Brood (T1)	3.70	1.50	0.17	− 0.12	0.48	0.38	0.33	0.42	0.36	–				
9. Brood (T2)	3.65	1.51	0.15	− 0.14	0.41	0.32	0.41	0.32	0.40	0.72	–			
10. Sleep (T1)	0.74	0.62	0.08	0.06**	0.35	0.30	0.29	0.31	0.28	0.35	0.31	–		
11. Sleep (T2)	0.76	0.62	0.07**	0.05*	0.31	0.26	0.34	0.26	0.31	0.30	0.37	0.71	–	
12. Confl (T1)	1.00	0.91	0.06**	− 0.12	0.33	0.49	0.39	0.53	0.42	0.37	0.29	0.22	0.18	–
13. Confl (T2)	1.00	0.92	0.08	− 0.13	0.28	0.39	0.48	0.39	0.51	0.30	0.37	0.22	0.24	0.52

Sex: men = 0, women = 1, Neuro = Neuroticism, NAQ = Exposure to bullying behaviours, HWC = Hostile work climate, Brood = Brooding, Sleep = Sleep problems, Confl = Conflict involvement

All correlations significant at *p* < .001 except where indicated: *p < .05, **p < .01, ns = not significant

**Fig. 1:**
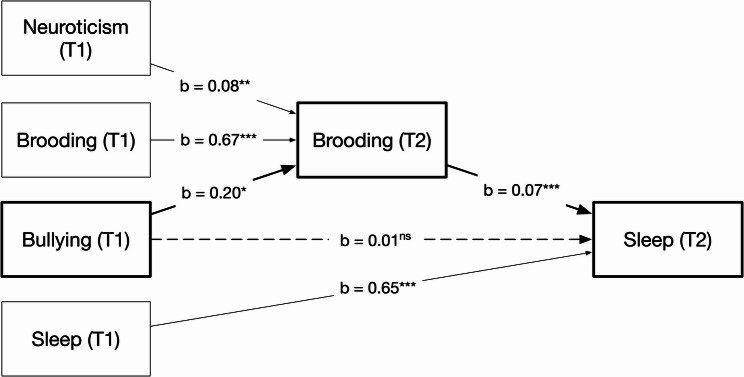
Mediation model showing the paths from exposure to bullying behaviours (T1) to sleep problems (T2) via brooding (T2). Note. Bullying = Negative Acts Questionnaire. Covariates: Sex, Age. Unstandardized coefficients. The dashed path is not statistically significant. *p < .05. **p < .01. ***p < .001. ns = not significant

As shown in Table 1, exposure to bullying behaviours was positively associated with sleep problems. To test Hypothesis 1, we examined whether brooding mediated this association. Controlling for sex, age, baseline neuroticism, brooding, and sleep problems, exposure to bullying behaviours at T1 predicted increased brooding at T2, *b* = 0.197, 95% BootCI [0.021, 0.386], *p* = .034, and brooding at T2 was associated with increased sleep problems at T2, *b* = 0.072, 95% BootCI [0.058, 0.087], *p* < .001 (see Fig. 1 and Table S1 in Supplementary Materials). The direct effect of bullying exposure on sleep problems was not significant, *b* = 0.011, 95% BootCI [-0.058, 0.087], *p* = .756, whereas the indirect effect via brooding was significant, *b* = 0.014, 95% BootCI [0.001, 0.028], *p* = .039, indicating that the association between bullying and sleep problems operated through brooding and supporting Hypothesis 1.

**Fig. 2:**
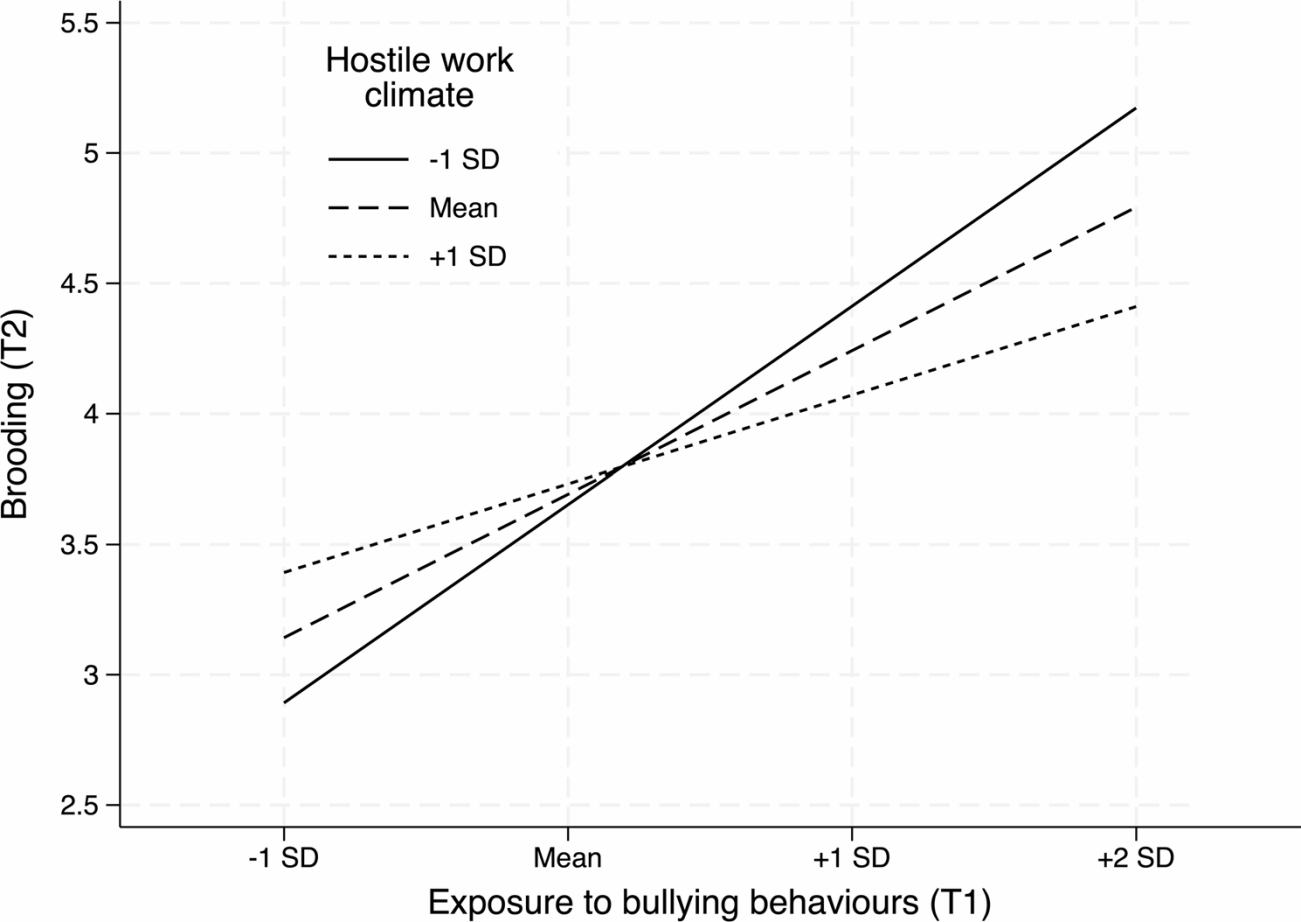
Interaction between exposure to bullying behaviours (T1) and hostile work climate (T1) in predicting brooding (T2). Note: Covariates: Sex, Age, Neuroticism (T1), and Brooding (T1)

To test Hypothesis 2, we examined whether the indirect effect depended on hostile work climate (HWC). As shown in Table 2, the interaction between exposure to bullying behaviours and HWC significantly predicted brooding at T2, *b* = -0.190, 95% BootCI [-0.317, -0.085], *p* < .001, indicating that the exposure–brooding association varied across work environments. Simple slopes showed that exposure predicted increased brooding at low HWC, *b* = 0.740, 95% BootCI [0.385, 1.126], *p* < .001, at the mean level of HWC, *b* = 0.523, 95% BootCI [0.256, 0.794], *p* < .001, and, more weakly, at high HWC, *b* = 0.306, 95% BootCI [0.089, 0.518], *p* = .005 (see Fig. 2). A Johnson–Neyman analysis indicated that the association between exposure to bullying behaviours and brooding was statistically significant across nearly the entire observed range of hostile work climate. The effect became non-significant only at the very highest levels of hostile work climate, corresponding to approximately the top 2.3% of the distribution. Consistent with this pattern, the conditional indirect effect via brooding was strongest at low HWC (-1 SD) *b* = 0.053, 95% BootCI [0.026, 0.084], *p* < .001, remained significant at mean HWC, *b* = 0.038, 95% BootCI [0.017, 0.060], *p* < .001, and was attenuated but still significant at high HWC (+ 1 SD), *b* = 0.022, 95% BootCI [0.006, 0.038], *p* = .007. The index of moderated mediation was significant, index = -0.014, *p* < .001, supporting Hypothesis 2.

**Table 2:** Moderated mediation model predicting brooding and sleep problems at T2

Predictor	b	Boot SE	95% BootCI	β	*p*
Brooding (T2)					
Exposure to bullying behaviours (NAQ, T1)	0.523	0.137	[0.256, 0.794]	0.102	< 0.001
Hostile work climate (HWC, T1)	-0.003	0.029	[-0.060, 0.053]	− 0.003	0.904
NAQ × HWC	-0.190	0.060	[-0.317, -0.085]	− 0.082	< 0.001
Brooding (T1)	0.666	0.020	[0.627, 0.704]	0.662	< 0.001
Neuroticism (T1)	0.077	0.028	[0.022, 0.134]	0.056	0.007
Sex	0.080	0.048	[-0.015, 0.173]	0.026	0.096
Age	-0.006	0.002	[-0.011, -0.002]	− 0.043	0.006
Sleep problems (T2)					
Brooding (T2)	0.072	0.007	[0.058, 0.087]	0.177	< 0.001
Exposure to bullying behaviours (NAQ, T1)	0.012	0.037	[-0.060, 0.083]	0.005	0.753
Sleep problems (T1)	0.649	0.019	[0.613, 0.685]	0.657	< 0.001
Sex	-0.008	0.019	[-0.046, 0.030]	− 0.006	0.673
Age	0.002	0.001	[0.001, 0.004]	0.040	0.009

NAQ = Exposure to bullying behaviours; HWC = Hostile work climate. Sex: men = 0, women = 1

**Fig. 3:**
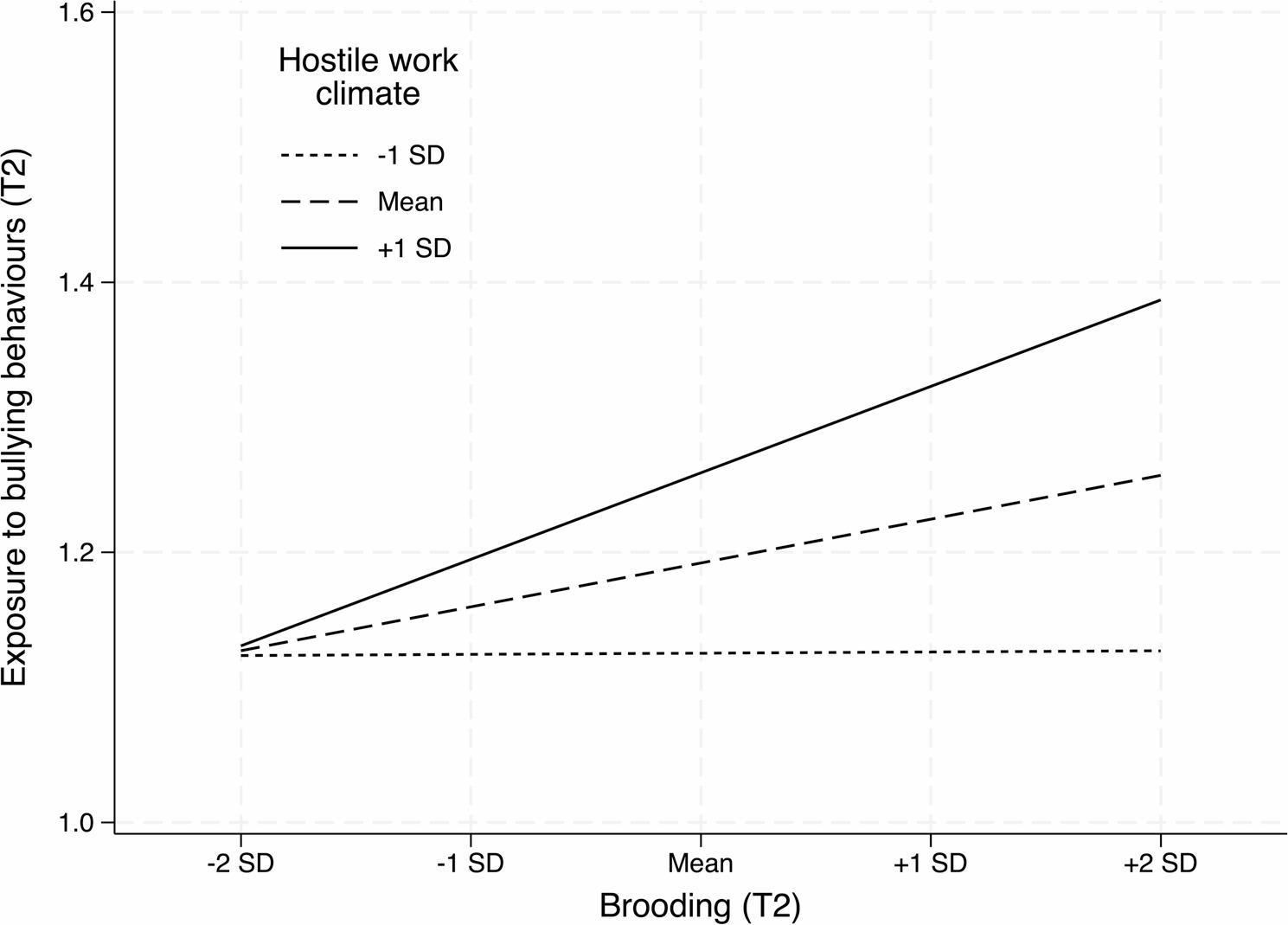
Interaction between brooding and hostile work climate in predicting exposure to bullying behaviours

Hypotheses 3 and 4 examined the reverse effect of sleep problems on subsequent exposure to bullying behaviours. Controlling for baseline exposure to bullying behaviours, brooding, conflict involvement, sex, age, and neuroticism, baseline sleep problems predicted increased exposure to bullying at follow-up through significant indirect effects via brooding, *b* = 0.022, 95% BootCI [0.015, 0.028], *p* < .001, and conflict involvement, *b* = 0.030, 95% BootCI [0.017, 0.042], *p* < .001. The direct effect of sleep problems on subsequent bullying was not statistically significant, *b* = 0.008, 95% BootCI [-0.008, 0.023], *p* = .332. Furthermore, the association between brooding and subsequent exposure to bullying was moderated by hostile work climate, *b* = 0.018, 95% BootCI [0.009, 0.029], *p* < .001 (see Table 3). Simple slopes showed that this association was significant at the mean level of HWC, *b* = 0.022, *p* < .001, and at high HWC, (+ 1 SD), *b* = 0.043, *p* < .001, but not at low HWC (-1 SD), *b* = 0.0004, *p* = .929 (see Fig. 3). The index of moderated mediation was significant, index = 0.002, *p* = .021, indicating a conditional indirect effect of sleep problems on subsequent bullying via brooding that was present at the mean level of HWC, *b* = 0.003, *p* < .003, and at high HWC, *b* = 0.006, *p* < .002, but not at low HWC.

**Table 3:** Mediation model predicting brooding, conflict involvement, and exposure to bullying behaviours at T2

Predictor	b	Boot SE	95% BootCI	β	*p*
Brooding (T2)					
Sleep problems (T1)	0.135	0.043	[0.048, 0.218]	0.056	0.002
Brooding (T1)	0.668	0.020	[0.629, 0.706]	0.664	< 0.001
Neuroticism (T1)	0.074	0.028	[0.019, 0.127]	0.054	0.008
Sex	0.083	0.048	[-0.010, 0.177]	0.027	0.084
Age	-0.008	0.002	[-0.012, -0.003]	− 0.054	0.001
Conflict involvement (T2)					
Sleep problems (T1)	0.175	0.030	[0.117, 0.236]	0.119	< 0.001
Conflict involvement (T1)	0.483	0.023	[0.436, 0.528]	0.478	< 0.001
Sex	0.088	0.034	[0.021, 0.155]	0.047	0.011
Age	-0.007	0.002	[-0.010, -0.004]	− 0.079	< 0.001
Bullying (T2)					
Sleep problems (T1)	0.008	0.008	[-0.008, 0.023]	0.017	0.332
Brooding (Brood, T2)	0.022	0.003	[0.015, 0.028]	0.113	< 0.001
Conflict involvement (Conflict, T2)	0.030	0.006	[0.017, 0.042]	0.094	< 0.001
Hostile work climate (HWC, T2)	0.058	0.006	[0.045, 0.070]	0.230	< 0.001
Brood×HWC	0.018	0.005	[0.009, 0.029]	0.127	< 0.001
Conflict×HWC	0.010	0.006	[-0.002, 0.022]	0.051	0.117
Bullying (T1)	0.443	0.037	[0.373, 0.516]	0.453	< 0.001
Sex	-0.010	0.008	[-0.025, 0.005]	− 0.017	0.202
Age	-0.000	0.000	[-0.001, 0.000]	− 0.020	0.199

NAQ = Exposure to bullying behaviours; HWC = Hostile work climate. Sex: men = 0, women = 1

In contrast, although conflict involvement also constituted a significant mediating pathway between sleep problems and subsequent bullying, the interaction between conflict involvement and hostile work climate was not significant. This indicates that the conflict pathway operated similarly across levels of hostile work climate. Together, these results supported Hypotheses 3a and 3b, as well as Hypothesis 4a, but not Hypothesis 4b.

To examine whether the inclusion of neuroticism influenced the results, additional analyses were conducted excluding neuroticism from all models. The overall pattern of results was essentially unchanged. Across all models, excluding neuroticism slightly increased the magnitude of the predictors of brooding, whereas the remaining paths were virtually identical. Full results from these analyses are reported in Supplementary Materials, Tables S2–S5.

## Discussion

The present study examined how workplace bullying and sleep are related by testing mechanisms that may explain both forward (bullying → sleep) and reverse (sleep → bullying) associations, as well as the role of the work environment. The results showed that exposure to bullying behaviours predicted increased subsequent sleep problems through brooding (Hypothesis 1), with this mechanism being most pronounced in seemingly friendly work environments (Hypothesis 2). Regarding the reverse association, sleep problems predicted subsequent exposure to bullying indirectly via both the conflict involvement and gloomy perception mechanisms (Hypotheses 3a and 3b). Moreover, the brooding-based indirect effect depended on the work environment and was present only under conditions of a hostile work climate (Hypothesis 4a).

The present findings suggest that sleep problems following exposure to workplace bullying are closely tied to how individuals attempt to make sense of what is happening at work. Although individuals differ in their general tendency to brood [27], the association was observed when controlling for neuroticism—the personality trait most consistently linked to brooding. This indicates that the process cannot be explained solely by dispositional differences. Additional analyses excluding neuroticism yielded a very similar pattern of results, although the associations predicting brooding became slightly stronger when neuroticism was omitted. This pattern suggests that controlling for neuroticism provided a more conservative estimate of the focal associations while alleviating concerns that the inclusion of this trait may have resulted in over-controlling. Instead, the findings indicate that brooding reflects a situational sense-making response to exposure to negative behaviours that are difficult to interpret, manage, and stop.

Negative social events at work are known to elicit affective reactions that shape subsequent cognitive processing [34]. When such events are repeated and perceived as ambiguous, unexpected, or unfair, sense-making efforts may intensify. In the context of workplace bullying—where behaviours often unfold gradually under conditions of power imbalance [2, 3]—these efforts may become increasingly constrained. Rather than reflective, problem-oriented pondering [29], cognitive processing may shift towards brooding, reflecting persistent attempts to understand situations in which perceived control is limited.

From this perspective, the finding that the indirect effect via brooding was strongest in seemingly friendly work environments is particularly informative. In such contexts, exposure to bullying behaviours may violate fundamental expectations of the workplace as a safe and predictable social environment, thereby creating cognitive dissonance. Experiences that are unexpected and difficult to reconcile with existing assumptions may challenge core beliefs about the world as coherent and predictable, intensifying efforts to make sense of what has occurred [47]. When such exposure is repeated and perceived as difficult to control or stop—conditions typical of bullying situations [2]—this process is likely to sustain cognitive activation during and beyond working hours [35], thereby interfering with recovery and sleep.

This pattern should not be interpreted as suggesting that hostile work environments are less harmful overall. On the contrary, hostile climates are associated with a higher risk of bullying and poorer job satisfaction and psychological well-being [45]. Rather, the present findings indicate that the *meaning* of bullying experiences—and the cognitive processes through which they affect sleep—varies depending on the broader work environment in which the exposure occurs.

Regarding the reverse effect, the present findings indicate that the association between sleep problems and subsequent exposure to bullying behaviours is more complex than previously assumed and operates indirectly rather than through a direct pathway. Previous studies have focused on direct effects and reported inconclusive results [4]. In contrast, the present findings suggest that this association is accounted for by two distinct indirect mechanisms.

The conflict involvement mechanism highlights how insufficient sleep may increase the risk of falling short of expected standards at work. When sleep problems manifest as tiredness, both task performance and social functioning may be impaired, increasing the likelihood of norm deviations and subsequent involvement in interpersonal conflicts. From a social interactionist perspective [53], such deviations may elicit corrective responses from others which, if poorly managed, may escalate into bullying behaviours. Support for this interpretation comes from Rosander and Nielsen [52], who showed that the association between tiredness at work and later exposure to bullying depended on how conflicts were managed, underscoring the role of conflict involvement as a linking mechanism.

In contrast, the gloomy perception mechanism suggests that sleep problems foster increased brooding. Heightened brooding reflects more extensive cognitive processing of work-related events, involving reduced regulation of emotional and attentional responses and a tendency to interpret ambiguous or mildly negative situations more negatively [54]. These cognitive changes may not only shape how situations are perceived but also increase tension or irritation in interactions with others, which—if poorly managed—may create conditions under which bullying behaviours are more likely to emerge [46].

Further support for this interpretation comes from the present findings showing that the indirect effect of sleep problems on subsequent exposure to bullying via brooding depended on the work environment. In hostile work climates, there is generally less capacity to address misunderstandings, frustration, or minor conflicts constructively, increasing the risk of escalation [45, 68]. Consistent with this, the indirect effect via brooding was confined to hostile work climates and was not observed in more well-functioning organizations. Previous research has shown that potential individual risk factors for bullying become less relevant in well-functioning organizational contexts [55]. A similar pattern was evident for the conflict involvement mechanism: although the interaction with hostile work climate was not statistically significant, hostile work climate itself was positively associated with exposure to bullying. This difference in contextual dependence between the two pathways may reflect the distinction between behavioural and cognitive processes. Conflict involvement represents observable behaviour that may elicit reactions from others regardless of the broader work environment. When interpersonal tensions or norm deviations become visible in everyday interactions, even generally well-functioning workplaces may respond in ways that escalate into negative treatment [46, 53]. In contrast, brooding represents a cognitive vulnerability that primarily shapes how situations are interpreted and emotionally processed. Such vulnerability may only translate into bullying when the surrounding work environment allows negative interpretations, tensions, or misunderstandings to develop into bullying behaviours [45, 68]. In more well-functioning environments, organizational norms and support may buffer such cognitive vulnerability before it escalates into bullying.

The findings indicate that sleep problems and exposure to bullying are closely interwoven through indirect, rather than direct, associations, with the work environment playing a central role. Exposure to bullying in seemingly friendly work environments was linked to increased brooding and subsequent sleep problems, whereas sleep problems increased the risk of bullying via brooding only in hostile work environments. In contrast, sleep problems associated with increased conflict involvement were linked to a higher risk of subsequent bullying regardless of the level of hostile work climate.

### Practical implications

The findings suggest that exposure to bullying may trigger persistent cognitive processing in the form of brooding, which in turn is associated with subsequent sleep problems. Importantly, this pathway was most pronounced in seemingly safe work environments, indicating that the absence of overt hostility does not preclude mechanisms through which bullying may disrupt sleep.

The results further suggest that sleep problems may increase vulnerability to bullying processes, particularly in hostile work environments, underscoring the importance of viewing impaired sleep as a contextual risk factor rather than solely an individual concern. Organizational capacity for early and constructive handling of interpersonal tensions appears critical, as even moderate conflict involvement associated with poor sleep was linked to an increased risk of bullying. These findings do not imply that sleep problems constitute an individual-level cause of bullying; rather, impaired sleep may heighten vulnerability in contexts where organizational conditions fail to buffer deviations from expected behaviour.

The present study focused on specific cognitive and contextual mechanisms linking exposure to bullying to sleep problems, and on how sleep-related impairments may, in turn, increase vulnerability to further bullying. While a wide range of other adverse outcomes of workplace bullying—such as impaired mental health, reduced well-being, and long-term health consequences—are well established (e.g., [69]), how sleep problems interact with these broader outcomes remains an important avenue for future research.

Overall, the findings indicate that the risk of developing sleep problems via brooding following exposure to bullying was most pronounced in seemingly safe work environments, whereas the translation of sleep-related cognitive vulnerability into increased bullying risk via brooding occurred only in hostile work environments. Rather than forming a uniform vicious cycle, these patterns suggest that contextual conditions may partly constrain reciprocal dynamics. Nevertheless, organizations should remain attentive to signs of sleep problems and their manifestations in everyday work behaviour, as early identification may help prevent bullying from emerging or escalating.

Organizational efforts to prevent bullying may also contribute to improved recovery, health, and work functioning among employees. Previous research has shown that factors such as perceived organizational support and a strong ethical climate can buffer the negative consequences of bullying [70]. By fostering supportive work environments with clear norms, fair leadership, and zero tolerance for mistreatment, organizations may reduce both the occurrence of bullying and its effects on sleep. Early intervention strategies—such as accessible reporting systems, manager training, and constructive conflict management—may further limit prolonged exposure to bullying and prevent the escalation of stress-related sleep problems.

In addition, initiatives that encourage leaders to recognize and model healthy sleep behaviours, such as incorporating sleep leadership into leadership training [71, 72], may reduce stress and fatigue that could otherwise contribute to bullying processes. By supporting leaders’ own recovery and functioning, such approaches may also foster work environments in which employees feel supported and better equipped to manage interpersonal challenges [73].

### Strength and limitations

A major strength of the present study is the use of a large longitudinal national probability sample drawn from the Swedish workforce. The longitudinal design enabled the examination of temporal associations between bullying, cognitive and behavioural mechanisms, and sleep problems in both directions. A further strength lies in the analytical strategy. All models were adjusted for baseline levels of mediators and outcomes, thereby focusing on change over time rather than static associations. Controlling for prior levels typically absorbs substantial variance through stability paths, often attenuating other effects. That the hypothesized mediation and moderation processes nevertheless emerged under these conservative conditions underscores the robustness of the findings.

Workplace bullying is a highly skewed phenomenon, as most individuals are not exposed. To address this non-normality, all models were estimated using bootstrapping with 5,000 resamples, an approach that reduces reliance on distributional assumptions and yields more reliable confidence intervals for indirect and conditional effects [67].

Several limitations should also be acknowledged. All measures relied on self-reports, which may raise concerns regarding subjective interpretations, social desirability, and common method variance [74]. However, the central constructs examined—exposure to bullying, brooding, conflict involvement, and sleep problems—are inherently subjective, and alternative “objective” indicators may introduce different sources of measurement error [15]. If social desirability influenced responses, it would likely have led to underreporting, resulting in more conservative rather than inflated estimates. In addition, the longitudinal design and the separation of key constructs across different sections of the questionnaire may have reduced the risk of common method variance [75]. A post hoc Harman’s single-factor test further indicated that common method variance is unlikely to account for the observed associations, as the first unrotated factor explained only 25% of the variance. Sleep problems were assessed using three items reflecting difficulties with sleep onset, sleep maintenance, and early morning awakening [58]. While these items capture core symptoms of insomnia, the measure is brief and its psychometric properties have been less extensively examined. It should also be noted that while exposure to bullying behaviours preceded brooding in the longitudinal design, brooding and sleep problems were measured at the same time point at follow-up. The association between brooding and sleep problems should therefore be interpreted as contemporaneous rather than strictly longitudinal. At the same time, cognitive activation related to rumination is generally assumed to influence sleep relatively quickly, making such temporal proximity theoretically plausible [35].

Finally, the forward and reverse associations examined may unfold over different time frames. Cognitive sense-making processes such as brooding may represent relatively early responses to ambiguous or stressful work situations [8, 47], whereas behavioural changes related to conflict involvement are more likely to emerge through repeated interactions and escalation over time [46, 53]. Future studies should therefore examine these mechanisms using different time lags to assess whether the observed patterns depend on temporal spacing. Future research could also investigate whether individuals with prior experiences of bullying respond differently to renewed exposure. Such experiences may shape cognitive scripts or heighten sensitivity, potentially amplifying brooding—particularly when negative acts occur in otherwise low-hostility work environments. These processes remain underexplored but may help explain why cognitive reactions to bullying vary across individuals.

## Conclusions

The present study contributes to the literature by clarifying how workplace bullying and sleep problems are related through specific cognitive and behavioural mechanisms, and by showing that these associations are context dependent. Exposure to bullying was linked to subsequent sleep problems through increased brooding, with this pathway being particularly pronounced in seemingly safe work environments. In contrast, sleep problems increased vulnerability to later bullying through brooding in more hostile work environments, while conflict involvement constituted a risk pathway irrespective of the level of hostile work climate.

Taken together, the findings suggest that the relationship between bullying and sleep is neither simple nor uniform. Rather than constituting a straightforward vicious cycle, the results indicate that different work environments intensify risk in different directions, which may partly attenuate the development of fully self-reinforcing reciprocal processes. By highlighting the role of cognitive sense-making and organizational context, the present study advances understanding of when and how sleep problems and workplace bullying become intertwined, and underscores the importance of considering both individual vulnerability and work environment conditions in future research and practice.

## Supplementary Information

Supplementary Material 1.

## Data Availability

The datasets used and/or analysed during the current study are available from the corresponding author on reasonable request.

## Abbreviations

AET: Affective events theory
CATS: Cognitive activation theory of stress
HWC: Hostile work climate
NAQ–R: Negative acts questionnaire–revised
