# Supplementary material for: Making Sense of Bullying: Brooding and subsequent sleep problems in seemingly safe work environments
Source: BMC Psychol. 2026 Apr 28;14:644. doi: 10.1186/s40359-026-04606-2 (PMC13137540; doi:10.1186/s40359-026-04606-2)
Supplement: Supplementary file 1 — Supplementary Material 1. [file 40359_2026_4606_MOESM1_ESM.pdf]

Supplementary Materials

Making Sense of Bullying: Brooding and Subsequent Sleep Problems in Seemingly Safe Work Environments

Michael Rosander & Morten Birkeland Nielsen

In all tables, unstandardized coefficients (b) with bootstrapped standard errors (Boot SE) and 95% bootstrap confidence intervals (BootCI) based on 5,000 resamples are reported. β represents completely standardized coefficients derived from the same model specification. Sex: men = 0, women = 1.

Table S1

Moderated mediation model predicting brooding and sleep problems at T2 (H1)

Predictor	*b*	Boot SE	95% BootCI	β	*p*
**Brooding (T2)**					
Exposure to bullying behaviours (T1)	0.197	0.093	[0.021, 0.386]	.038	.035
Brooding (T1)	0.671	0.019	[0.633, 0.708]	.666	< .001
Neuroticism (T1)	0.086	0.028	[0.031, 0.139]	.062	.002
Sex	0.079	0.047	[-0.012, 0.172]	.026	.098
Age	-0.007	0.002	[-0.011, -0.002]	-.046	< .001
**Sleep problems (T2)**					
Brooding (T2)	0.072	0.007	[0.058, 0.087]	.177	< .001
Exposure to bullying behaviours (T1)	0.011	0.037	[-0.058, 0.087]	.005	.756
Sleep problems (T1)	0.649	0.019	[0.612, 0.685]	.656	< .001
Sex	-0.008	0.019	[-0.045, 0.031]	-.006	.684
Age	0.002	0.001	[0.001, 0.004]	.040	.009

Sensitivity analyses excluding neuroticism (Table S2–5). To examine whether the inclusion of neuroticism influenced the results, all models were re-estimated without neuroticism. The overall pattern of results remained unchanged, although the predictors of brooding became slightly stronger.

Table S2

Moderated mediation model predicting brooding and sleep problems at T2 (H1)

Predictor	*b*	Boot SE	95% BootCI	β	*p*
**Brooding (T2)**					
Exposure to bullying behaviours (T1)	0.239	0.090	[0.065, 0.417]	.047	.008
Brooding (T1)	0.695	0.018	[0.662, 0.730]	.691	< .001
Sex	0.098	0.047	[0.007, 0.192]	.032	.038
Age	-0.008	0.002	[-0.012, -0.004]	-.056	< .001
**Sleep problems (T2)**					
Brooding (T2)	0.072	0.008	[0.057, 0.087]	.178	< .001
Exposure to bullying behaviours (T1)	0.018	0.037	[-0.055, 0.091]	.009	.616
Sleep problems (T1)	0.648	0.018	[0.612, 0.685]	.656	< .001
Sex	-0.008	0.019	[-0.047, 0.030]	-.006	.679
Age	0.002	0.001	[0.001, 0.004]	.040	.011

Table S3
Moderated mediation model predicting brooding and sleep problems at T2 (H2)

Predictor	*b*	Boot SE	95% BootCI	β	*p*
**Brooding (T2)**					
Exposure to bullying behaviours (NAQ, T1)	0.561	0.130	[0.308, 0.819]	.110	< .001
Hostile work climate (HWC, T1)	0.006	0.028	[-0.048, 0.061]	.005	.819
NAQ × HWC	-0.206	0.058	[-0.332, -0.104]	-.088	< .001
Brooding (T1)	0.686	0.018	[0.650, 0.722]	.682	< .001
Sex	0.096	0.047	[0.006, 0.190]	.031	.040
Age	-0.008	0.002	[-0.012, -0.003]	-.052	.001
**Sleep problems (T2)**					
Brooding (T2)	0.072	0.007	[0.058, 0.087]	.178	< .001
Exposure to bullying behaviours (NAQ, T1)	0.018	0.038	[-0.055, 0.093]	.009	.623
Sleep problems (T1)	0.648	0.019	[0.611, 0.686]	.656	< .001
Sex	-0.008	0.019	[-0.045, 0.030]	-.007	.667
Age	0.002	0.001	[0.001, 0.004]	.040	.009

Table S4
Mediation model predicting brooding, conflict involvement, and exposure to bullying behaviours at T2 (H3)

Predictor	*b*	Boot SE	95% BootCI	β	*p*
**Brooding (T2)**					
Sleep problems (T1)	0.164	0.042	[0.084, 0.248]	.068	< .001
Brooding (T1)	0.688	0.018	[0.651, 0.723]	.684	< .001
Sex	0.100	0.047	[0.007, 0.194]	.032	.035
Age	-0.009	0.002	[-0.014, -0.005]	-.064	< .001
**Conflict involvement (T2)**					
Sleep problems (T1)	0.175	0.030	[0.115, 0.232]	.119	< .001
Conflict involvement (T1)	0.484	0.023	[0.438, 0.529]	.479	< .001
Sex	0.089	0.035	[0.022, 0.159]	.048	.011
Age	-0.007	0.002	[-0.010, -0.004]	-.078	< .001
**Bullying (T2)**					
Sleep problems (T1)	0.018	0.009	[-0.001, 0.034]	.038	.044
Brooding (T2)	0.028	0.004	[0.021, 0.036]	.148	< .001
Conflict involvement (T2)	0.064	0.007	[0.049, 0.078]	.202	< .001
Bullying (T1)	0.546	0.040	[0.471, 0.629]	.560	< .001
Sex	-0.002	0.008	[-0.018, 0.014]	-.003	.816
Age	-0.000	0.000	[-0.001, 0.001]	-.006	.735

Table S5
Mediation model predicting brooding, conflict involvement, and exposure to bullying behaviours at T2 (H4)

Predictor	*b*	Boot SE	95% BootCI	β	*p*
**Brooding (T2)**					
Sleep problems (T1)	0.164	0.042	[0.082, 0.248]	.068	< .001
Brooding (T1)	0.688	0.018	[0.651, 0.723]	.684	< .001
Sex	0.100	0.047	[0.007, 0.192]	.032	.035
Age	-0.009	0.002	[-0.014, -0.005]	-.064	< .001
**Conflict involvement (T2)**					
Sleep problems (T1)	0.175	0.030	[0.115, 0.232]	.119	< .001
Conflict involvement (T1)	0.484	0.023	[0.438, 0.529]	.479	< .001
Sex	0.089	0.035	[0.022, 0.159]	.048	.011
Age	-0.007	0.002	[-0.010, -0.004]	-.078	< .001
**Bullying (T2)**					
Sleep problems (T1)	0.008	0.008	[-0.008, 0.024]	.017	.319
Brooding (Brood, T2)	0.021	0.003	[0.015, 0.028]	.112	< .001
Conflict involvement (Conflict, T2)	0.030	0.006	[0.017, 0.043]	.095	< .001
Hostile work climate (HWC, T2)	0.058	0.006	[0.045, 0.070]	.231	< .001
Brood×HWC	0.018	0.005	[0.008, 0.029]	.126	< .001
Conflict×HWC	0.010	0.006	[-0.003, 0.022]	.050	.127
Bullying (T1)	0.440	0.037	[0.369, 0.513]	.452	< .001
Sex	-0.010	0.008	[-0.025, 0.005]	-.017	.194
Age	-0.001	0.000	[-0.001, 0.000]	-.020	.202
